# Suicide Attempts by Poisoning: An Experience From a High-Volume Emergency Department

**DOI:** 10.7759/cureus.23330

**Published:** 2022-03-20

**Authors:** Faisal K Alrasheed, Yazeed A Alowairdhi, Yasser M Alkharashi, Abdulrahman O Alomar, Muhannad Q Alqirnas, Nawaf A Alhussaini, Abdulrahman Albassam, Abdulaziz S Almosa, Ahmed Z Alkhars, Mohammed Alhelail

**Affiliations:** 1 College of Medicine, King Saud Bin Abdulaziz University for Health Sciences, Riyadh, SAU; 2 Pediatrics, College of Medicine, King Faisal University, Al-Ahsa, SAU; 3 Emergency Department, King Abdulaziz Medical City, Riyadh, SAU; 4 Emergency Department, King Abdullah International Medical Research Center, Riyadh, SAU

**Keywords:** toxicology, saudi arabia, suicidology, suicide, poisoning, emergency medicine

## Abstract

Background

There has been a tremendous increase in self-poisoning behavior worldwide, with different trends depending on cultural and geographic aspects.

Objectives

Our study aims to assess the trends, outcomes, and predictors in patients of suicide attempts by poisoning at King Abdulaziz Medical City (KAMC) ED.

Materials and methods

A retrospective cohort study took place at KAMC. Frequencies and percentages were used to display categorical variables. Minimum, maximum, mean, and SD were used to display continuous variables. Chi-squared test and independent t-test were utilized to test for factors associated with suicidal intention.

Results

A total of 130 cases were identified. The participants were mostly females (73.8%, n = 96). Most of the participants were pediatric patients (57.7%, n = 75). The most consumed agents were acetaminophen in 59 (45.83%) and non-steroidal anti-inflammatory drugs (NSAIDs) in 22 (16.92%). The ICU admission rate was 8.5% (n = 11). The management for both populations was unspecific, involving observation, supportive measures, and symptomatic treatment. BMI (*p* < 0.001), gender (*p* < 0.001), age (*p *= 0.012), and a history of neuropsychiatric disorders (*p* < 0.001) were associated factors.

Conclusion

It is crucial that the trends and risk factors of self-poisoning suicide attempts are identified to provide support to those in need. Several variables of interest were noted since the two most observed agents share several key features, such as accessibility and availability. However, contradicting literature reports warrant further investigation to confirm or negate the evidence.

## Introduction

Suicide is the act of intentionally causing one's own death [1]. Many factors have been linked with suicide. Some of these factors include substance use disorder and mental disorders [2]. Various methods are used to commit suicide, such as hanging, the use of firearms, and self-poisoning [3-5]. It can be categorized as non-violent (drugs and poisons) and violent methods [3]. Differences in suicide means are observed across different demographical groups. For example, self-poisoning is one of the most common suicidal mechanisms in all groups, and it is also the most common one in young females [3,5,6]. On the other hand, violent methods are more common in males and the mentally ill [3]. Also, rural community members are more likely to suicide using a firearm than urban community members [7].

Intentional self-poisoning, the non-violent method of suicide, occurs most commonly through the utilization of medication, either prescribed (such as antidepressants and prescription analgesics) or over-the-counter (paracetamol), chemical agents (pesticides), or illicit drugs [3]. Cases of illicit drug self-poisoning might have difficulty determining whether they were intentional or not [3]. Antidepressants followed by analgesics accounted for the major number of ED poisoning cases as reported in the literature [8,9]. However, analgesics, especially acetaminophen, accounted for the major part of suicide attempts through self-poisoning [10-11]. In addition, a nation-wide American study found an increase of self-poisoning suicide attempts during school months in adolescents [11].

There has been a tremendous increase in self-poisoning behavior worldwide, especially among adolescents and young adults. These individuals have a higher risk of repeating such behavior in the future [12]. Therefore, it is important to study and identify suicide methods and the groups at risk of suicide, making these methods less accessible and preventable [13-15]. Moreover, the early identification of neuropsychiatric and substance abuse disorders and the proper follow-up care for those who have attempted suicide play an important role in decreasing the number of deaths by suicide [16]. Thus, our study aims to assess the trends, outcomes, and predictors in patients of suicide attempts by poisoning at King Abdulaziz Medical City (KAMC) ED.

## Materials and methods

Study design, settings, and inclusion criteria

This study was a retrospective cohort study conducted at King Abdulaziz Medical City (KAMC) for patients aged more than 14 years and King Abdullah Specialized Children Hospital (KASCH) for the patients aged 14 and less in Riyadh, Saudi Arabia. The inclusion criteria for this study were all patients presenting with acute poisoning that resulted from an intentional suicide attempt from January 1, 2016, to January 1, 2021. A total of 1505 patients presented to the ED with acute poisoning during that period. Among them, 130 cases were due to a suicidal intention. These patients were identified and included if the history of the presenting complaint indicated a suicide attempt.

Data collection

The patients' records were retrospectively tracked, and data were collected from them. Collected data included demographics, medical history, poisoning agents, length of stay, discharge type, either a complete discharge or a premature discharge, which often happens if the hospital is overcrowded or understaffed. We also gathered the presenting complaints, types of intervention provided, and outcomes. The parameters were obtained from the BESTCare system (ezCareTech, South Korea). For example, if a patient had an ICU admission, organ transplantation, and mortality, they were classified as poor outcomes. Any outcomes that differed from the three aforementioned outcomes were labeled as good outcomes.

Statistical analysis

Statistical analysis was done using the SPSS version 23.0 (IBM Corporation, NY, USA). Frequencies and percentages were used to display categorical variables. Minimum, maximum, mean, and SD were used to display continuous variables. Chi-squared test and independent t-test were utilized to test for factors associated with suicidal intention. In order for the test of association to be carried on and to determine factors associated with suicidal poisoning, the initially excluded group (patients who presented to the ER as a case of poisoning for a reason other than suicide) were compared to patients.

Ethics

Ethical approval with the number RC20/676/R was obtained from the Institutional Review Board of King Abdullah International Medical Research Center. Patient confidentiality was ensured, and the patient data were collected and used only by the research team. Due to the study's retrospective nature and the use of anonymized patient data, the requirement for informed consent was waived.

## Results

A total of 1505 poisoning cases were identified in the records. Among them, only 130 were suicide cases and thus were included in the study. The study population was predominantly composed of males (73.8%, n = 96) regarding the demographic profile. When it comes to age groups, most of the participants were classified as pediatric patients (57.7%, n = 75) aged less than 18. Furthermore, most patients were not smokers (93.1%, n = 121). As for the BMI status, most patients had normal weight (37.7%, n = 49).

The patient population predominantly had no comorbidities (71.5%, n = 93). However, the most observed medical conditions were neuropsychiatric disorders in 26 (20%), cardiovascular diseases in six (4.6%), diabetes mellitus in four (3.1%), renal disease in three (2.3%), cancer in two (1.5%), and nine patients (7.2%) had other miscellaneous comorbidities.

Table 1 displays the poisoning history of patients. As for the number of poisoning agents consumed by the patients, 79 (60.8%) consumed one agent, and 51 (39.23%) had multiple drug ingestion. A total of 34 (26.2%) consumed two agents, nine (6.9%) consumed three agents, five (3.8%) consumed four agents, one (0.8%) consumed five agents, one (0.8%) consumed six agents, and one (0.8%) consumed nine agents. As for the premature discharge, only three (2.3%) were prematurely discharged, while 127 (97.7%) were not. As for the patients' outcomes, 119 (91.5%) had good outcomes, and 11 (8.5%) had ICU admission. As for the length of stay, the mean length was 2.06 + 2.89 days. The minimum was four hours (0.17 days), and the maximum was 29 days.

**Table 1:** Poisoning history.

Poisoning History (n = 492)	n	%
Number of poisoning agents		
1	79	60.8
2	34	26.2
3	9	6.9
4	5	3.8
5	1	0.8
6	1	0.8
9	1	0.8
Premature discharge		
No	127	97.7
Yes	3	2.3
Outcome		
Good	119	91.5
ICU admission	11	8.5
Length of stay (in days)		
Mean	2.06	
SD	2.89	
Minimum	0.17	
Maximum	29	

Table 2 demonstrates the agents of poisoning consumed by the participants. The most consumed agents were acetaminophen in 59 (45.83%), non-steroidal anti-inflammatory drugs (NSAIDs) in 22 (16.92%), antibiotics in 13 (10%), anticonvulsants in 11 (8.46%), antihistamine in 10 (7.69%), atypical antipsychotic in nine (6.92%), antidiabetic in eight (6.15%), and selective serotonin reuptake inhibitors in eight (6.15%).

**Table 2:** Ingested agents.

Ingested Agents	n	%	Ingested Agents		n	%
Acetaminophen	59	45.38	Estrogen		1	0.77
Amphetamine	1	0.77	Guaifenesin		2	1.54
Antibiotic	13	10.00	Herbal		2	1.54
Anticholinergic	1	0.77	Immunosuppressant		1	0.77
Anticoagulant	2	1.54	Laxative		1	0.77
Anticonvulsant	11	8.46	Minerals		7	5.38
Antidepressant	1	0.77	Miscellaneous		3	2.31
Antidiabetic	8	6.15	Muscle relaxant		5	3.85
Antiemetic	2	1.54	Non-steroidal anti-inflammatory drugs		22	16.92
Antihistamine	10	7.69	Organophosphate		2	1.54
Antiplatelet	1	0.77	Proton pump inhibitor		5	3.85
Antithyroid	1	0.77	Pseudoephedrine		3	2.31
Atypical antipsychotic	9	6.92	Salicylate		6	4.62
Beta2-agonist	1	0.77	Simethicone		1	0.77
Beta-blocker	2	1.54	Selective serotonin reuptake inhibitors		8	6.15
Benzodiazepine	2	1.54	Statin		3	2.31
Calcium channel blockers	1	0.77	Steroid		1	0.77
Central nervous system stimulant	2	1.54	Tricyclic anti-depressants		1	0.77
Dextromethorphan	2	1.54	Unknown agent		2	1.54
Disinfectant	5	3.85	Vitamins		5	3.85
Diuretic	1	0.77				

Table 3 illustrates the key interventions received by the participants during their admission. The majority of the patients, 101 (77.69%), received observation, supportive management, and ward admission. Eight (6.15%) patients received decontamination and elimination therapy, with activated charcoal being the most used intervention for decontamination and elimination therapy in six patients (4.62%). Among the patients, 28 (21.54%) had antidotal and targeted therapy, with N-acetylcysteine being the most common intervention used as antidotal and targeted therapy (n = 14, 10.77%). One (0.77%) of the patients received blood/blood products (specifically fresh frozen plasma) while seven (5.38%) received miscellaneous interventions: intubation in two (1.54%). Among the patients, five (3.85%) did not have a documented intervention.

**Table 3:** Key interventions received by the participants during their admission.

Intervention	n	%
Observation, supportive management, and admission		
Total	101	77.69
Admission	12	9.23
Observation and reassurance	65	50.00
Supportive and symptomatic management	24	18.46
Decontamination and elimination		
Total	8	6.15
Activated charcoal	6	4.62
Decontamination	1	0.77
Gastric lavage	1	0.77
Lipid emulsion	1	0.77
Antidotal and targeted therapy		
Total	28	21.54
Benzodiazepine	2	1.54
Benztropine (anticholinergics)	1	0.77
Deferoxamine	1	0.77
Ketamine	1	0.77
Midazolam	2	1.54
N-Acetylcysteine	14	10.77
Naloxone	2	1.54
Proton pump inhibitor	2	1.54
Ranitidine	1	0.77
Sodium Bicarbonate	1	0.77
Vitamin K	1	0.77
Blood and blood products		
Total	1	0.77
Fresh frozen plasma	1	0.77
Miscellaneous		
Total	7	5.38
Intubation	2	1.54
Not documented	5	3.85

Table 4 shows the clinical presentation of patients. A total of 28 (21.54%) of the patients were asymptomatic. The most observed clinical presentations were vomiting in 43 (33.08%), abdominal pain in 36 (27.69%), altered level of consciousness in 23 (17.69%), dizziness in 15 (11.54%), and nausea in 12 (9.23%). 

**Table 4:** Presenting complaints of poisoning.

Presenting Complaints of Poisoning (n = 130)	n	%
Clinical presentation		
Abdominal pain	36	27.69
Agitation	4	3.08
Altered level of consciousness	23	17.69
Asymptomatic	28	21.54
Bleeding	1	0.77
Chest pain	5	3.85
Diaphoresis	1	0.77
Diarrhea	1	0.77
Dizziness	15	11.54
Dystonic reaction (dyskinesia)	1	0.77
Generalized weakness (lethargy)	2	1.54
Hallucinations	2	1.54
Headache	6	4.62
Nausea	12	9.23
Palpitation (tachycardia)	8	6.15
Paresthesia	2	1.54
Rash	1	0.77
Restless	1	0.77
Shivering	2	1.54
Shortness of breath	5	3.85
Tinnitus	1	0.77
Tremor	1	0.77
Urine incontinence	1	0.77
Visual disturbance (diplopia)	3	2.31
Vomiting	43	33.08
Not documented	1	0.77

Table 5 displays the factors associated with suicide in poisoning cases. For the sake of determining the factors associated with suicide attempts, the 1505 cases of poisoning were included in this table. BMI was significantly associated with suicide in poisoning cases (p < 0.001), where those attempting suicide had a significantly higher BMI mean compared to those who did not attempt suicide (22.39 + 8.94 vs. 19.33 + 7.42). Gender was also significantly associated with suicide attempts in poisoning cases (p < 0.001), where females had a higher rate of suicide in poisoning cases compared to males (13.7% vs. 4.1%). The age group was also significantly associated with suicide attempts in poisoning cases (p < 0.012), where adults had a significantly higher rate of suicide attempts in poisoning cases compared to the pediatric age group (11.2% vs. 7.3%). Neuropsychiatric disorders were also significantly associated with suicide attempts in poisoning cases (p < 0.001), where those with neuropsychiatric disorders had a significantly higher rate of suicide in poisoning cases compared to those who do not have neuropsychiatric disorders (20.5% vs. 7.5%). A number of comorbidities and having a chronic disease were not significantly associated with suicide attempts in poisoning cases.

**Table 5:** Factors associated with suicide using poisoning. * represents p < 0.05.

Factors Associated with Suicide Using Poisoning				
Factor		Suicide		P-Value
		Yes	No	
BMI (mean, SD)		22.39 + 8.94	19.33 + 7.42	< 0.001*
Number of comorbidities (mean, SD)		1.1 + 0.41	1.15 + 0.58	0.347
Gender				< 0.001*
Male		33 (4.1%)	770 (95.9%)	
Female		96 (13.7%)	606 (86.3%)	
Age group				0.012*
Pediatric		74 (7.3%)	939 (92.7%)	
Adults		55 (11.2%)	437 (88.8%)	
Medical history				0.267
Have a chronic disease		37 (10%)	334 (90%)	
Medically free		92 (8.1%)	1042 (91.9%)	
Neuropsychiatric disorders				<0.001*
Yes		26 (20.5%)	101 (79.5%)	
No		103 (7.5%)	1275 (92.5%)	
*Significant at level 0.05				

## Discussion

The most observed clinical presentations were vomiting in 33.08%, abdominal pain in 27.69%, altered level of consciousness in 17.69%, dizziness in 11.54%, and nausea in 19.23%, while 21% of the patients were asymptomatic. These are all characteristic presentations of poisoning patients and predictors of poisoning-related fatalities [17].

Our study showed that 14% of females presenting to our ED due to poisoning were suicidal. This percentage was significantly higher when compared with males, which were only 4%. This is substantiated by Rajapakse T et al., that conducted a comparison study in Sri Lanka over a 14-month period to describe the gender differences in non-fatal self-poisoning. The findings here highlight that the patterns of non-fatal self-poisoning indicate a greater incidence of both self-poisoning and medicinal overdoses in females when compared with males [18]. An additional study by Tsirigotis K et al. further corroborated this in a study to assess the gender differentiation in methods of suicide attempts, highlighting that in a cohort of 234, all suicide attempts by poisoning were carried out by females [19]. However, a study conducted in Southern India by Kanchan T et al. contradicted these findings in a retrospective assessment that aimed to examine the difference in the pattern of suicidal poisoning among both males and females. This five-year study noted a significantly higher (73.9%) incidence of male suicides by poisoning when compared to females (26.3%) in a cohort of 137 that died because of suicidal poisoning. Despite this, the study does highlight that of the total cohort assessed (762 patients), a higher proportion of female patients were suffering from depression (27.8%) when compared to male patients (10.9%) [20].

When looking at the BMI status, those with higher BMI had a higher incidence of suicide in our study. However, this is not well reported in the literature, and the investigations substantiating these findings are scarce. A systematic review by Perera S et al. represents the most up-to-date collation of the literature on this variable and its relationship with suicide behaviors. This review, however, does not support our findings that a higher BMI may result in a higher incidence of suicide, with the current literature suggesting an inverse relationship. In an assessment of 38 observational studies, Perera S et al. highlight that an inverse association between BMI and completed suicide exists. A pooled summary suggests that a significantly low BMI is associated with an increased risk of suicide. While being obese and overweight were significantly associated with a reduced risk of suicide compared to those of a normal BMI [21].

Notably, non-smokers had a higher incidence of suicide attempts when compared to smokers. The demographic profile of the cohort included in our study revealed that 93.1% of the patients presenting with a case of poisoning were non-smokers. However, as with BMI, this variable is not widely discussed as a risk factor to suicide; hence, our observation here may only be a coincidence. This is also suggested in the contradictory findings of the current literature. A meta-analysis by Poorolajal J and Darvishi N concluded that when compared to non-smokers, smokers were at an enhanced risk of suicidal ideation, suicide planning, suicide attempt, and suicide death in a cohort of over eight million participants spanning 63 studies [22]. This negates our findings that suggest non-smokers are at enhanced risk of suicide, with the evidence in the current literature suggesting the opposite; being a smoker is a risk factor for suicide.

The majority of our cohort who attempted suicide had no comorbidities (71.5%, n = 93), and in those with comorbidities, patients with neuropsychiatric disease represent the highest proportion (20%). These findings warrant further investigation as our results are not conclusive, and the current literature is scarce. The outcomes of the suicide attempts in our study, on the other hand, are in concordance with the previous international published reports except for the use of antibiotics. ICU admission was necessary for 9% of patients assessed, with admission duration being an average of two days. Moreover, the most consumed agents were acetaminophen, NSAIDs, and antibiotics. Antibiotics are rarely reported as a suicide agent; however, the current literature does suggest that exposure to several antibiotics may be associated with suicidal behavior [23].

Despite the data discussed in this study and the current literature, the under-reporting of suicide represents a global challenge. This is emphasized in several studies, including Li F and Yip PS, that discussed the severe under-reporting of the national suicide rates published by the Chinese Ministry of Health [24]. Tøllefsen IM et al. also reported on this in a systematic review that corroborated the general under-reporting of suicide. This review also recommended that nationwide studies and comparisons be conducted between countries to comprehend the rationale for this oversight [25]. The under-reporting of suicide across the globe represents a recurrent limitation and hinders the external validity of not only our findings but also that of other studies evaluating the incidence of suicide.

The primary strength of our study is that, to the best of our knowledge, this represents one of the first investigations that discusses the topic of suicide attempts by poisoning in Saudi Arabia, with a relatively large sample size. Moreover, our study was conducted in a single large tertiary care center; hence, more patients were included in this study than other investigations focusing on primary care centers. However, there are several limitations of note. The study design being a retrospective chart review represents the primary limitation. Moreover, being conducted in only a single center. This introduces several types of bias that must be considered. Further studies should adopt a prospective approach and include more than one center to reduce bias within the findings and enhance the overall credibility of the results.

## Conclusions

The incidence of poisoning as a method of suicide is increasing, with the presentation of this at the ED being characteristic. However, it is crucial that the risk factors to self-poisoning and suicide attempts are identified to support those who require it. Our study noted several variables of interest, including being female, a high BMI, non-smokers, being medically free, and having a neuropsychiatric disease. Although some of our findings are contradicted in the current literature, they warrant further investigation in future studies to confirm or negate the evidence provided in this study.
